# Access to therapy for child sexual abuse survivors: Preliminary dialogue of barriers and facilitators between caregivers

**DOI:** 10.1371/journal.pone.0294686

**Published:** 2023-11-17

**Authors:** Jonathan Jin, Huda Al-Shamali, Lorraine Smith-MacDonald, Matthew Reeson, Wanda Polzin, Yifeng Wei, Hannah Pazderka, Peter H. Silverstone, Andrew J. Greenshaw

**Affiliations:** 1 Department of Psychiatry, University of Alberta, Edmonton, Canada; 2 Heroes in Mind Advocacy and Research Consortium, Faculty of Rehabilitation Medicine, University of Alberta, Edmonton, Alberta, Canada; 3 Little Warriors Be Brave Ranch, Edmonton, Alberta, Canada; Federal Ministry of Agriculture and Rural Development, NIGERIA

## Abstract

**Background:**

Difficulties in access to therapy were highlighted by COVID-19 measures restricting in-person gatherings. Additional challenges arise when focusing on caregivers of child sexual abuse (CSA) survivors in particular, which are a population that has been historically difficult to engage with due to issues of stigma and confidentiality.

**Objectives:**

To present preliminary qualitative results from caregivers of CSA survivors.

**Methods:**

This study was conducted with caregivers of CSA survivors. Two hybrid webinar/focus groups were conducted using a video conferencing platform in fall of 2021 with two groups of stakeholders (11 caregivers and 5 moderators/clinical staff at Little Warriors, an intensive episodic treatment facility). Sessions were recorded, transcribed, and thematically-analyzed using standard qualitative methodology.

**Results:**

A total of 11 caregivers contributed to the data. Themes include: (1) Challenges of starting and maintaining treatment (i.e., emotional impact of intake day, challenges of enrolling), (2) Therapeutic benefits of specialized treatment (i.e., feeling safe and supported and the importance of trauma-informed care), and (3) Barriers and facilitators of treatment (i.e., avenues to scale-up and self-care).

**Conclusion:**

The importance of a strong therapeutic alliance was highlighted by both caregivers/clinical staff and further support is needed for families post-treatment. The present hybrid webinar/focus group also achieved engagement goals in a population that is typically difficult to reach. Overall, the response rate (12%) was equivalent to reported registrant attendance rates for general business to consumer webinars and the recommended focus group size. This preliminary approach warrants replication in other populations outside our clinical context.

## Introduction

Millions of children globally have been survivors of child sexual abuse (CSA)—according to the World Health Organization’s 2022 “World report on violence and health”, the lifetime prevalence of CSA is 20% for women and 8% for men [1]. CSA survivors are at a higher risk of experiencing mental health challenges following the abuse. Such challenges include the development of posttraumatic stress disorder (PTSD), suicidal ideation and attempts, increased healthcare utilization, and engaging in health-risk behaviours [2, 3].

Accessing effective and specialized treatment for the unique needs of CSA survivors is important. However, many cases of CSA go undisclosed and therefore untreated. A reluctance to disclose can arise from a multitude of reasons ranging from feelings of guilt or a belief they will not be believed due to fear of stigma and negative cultural outlooks [4, 5]. Caregivers play an essential role in facilitating care for CSA survivors and previous studies have shown multiple levels of barriers to accessing mental health services [6].

For CSA survivors, access to therapy is challenging due to stigma (i.e., abuse occurring when the caregiver was the legal guardian [7]). Trust is particularly important in engaging CSA survivors and their families and protection of anonymity of children and caregivers is important [8, 9], in alignment with a trauma-informed approach [10]. We were interested in getting feedback (in the form of caregivers’ attitudes and opinions) about the Little Warriors treatment facility for CSA survivors. To address this, we adopted a novel engagement approach using a hybrid webinar/focus group strategy with video conferencing technology (building on previous work: [11, 12]). By involving two stakeholder groups (caregivers and treatment facility staff), as suggested by previous systematic reviews, we developed an efficient and ethical way to engage caregivers of CSA survivors and generate value for stakeholders in an anonymous question and answer (Q&A) format [13]. The conceptualization of this approach arose during discussions around the challenges in engaging with vulnerable populations, such as CSA survivors, and COVID-19 public health measures. Our research team first considered piloting a hybrid webinar/focus group approach to stimulate engagement. This option was deemed attractive as it may offer value on caregivers in relation to clinical program information. Given a voluntary choice to participate in a hybrid webinar/focus group, this approach was considered as it also facilitated engagement as opposed to being coercive.

The hybrid approach, which combines webinar and focus group elements, utilizes video conferencing technology, making it convenient and accessible for caregivers to participate from their own homes or locations of their choice. This eliminates the need for physical travel and reduces the barriers associated with in-person meetings. It also allows participants to join the sessions using anonymous profiles, protecting their identity and providing a sense of safety. This anonymity can encourage caregivers to share their experiences and opinions more openly, as they may feel more comfortable discussing sensitive or stigmatized issues.

A similar approach was used with university students [14], but it has not been investigated in clinical populations. Deeb-Sossa and colleagues [14] aimed to diversify the college admissions applicant pool at the University of California, Davis. Their results suggest use of a virtual webinar format introduces students as well as their families to campus life helps minority groups making enrollment decisions, especially during COVID-19.

When implementing this type of approach, it is important to consider some general statistics around webinar attendance in the general public domain. In accord with several public domain sources, a recent report of business webinar statistics indicates that typically an average of 260 individuals registers for a webinar but only 40–50% people may attend, and 78% of business to consumer webinars have ≤ 50 people in attendance [15]. Given the vulnerable nature of our CSA community and our experience of consulting with treatment facility stakeholders, we considered *a priori* that an attendance of ≥ 15% of registrants attending would be a successful outcome. With this goal in mind, we hypothesized that a hybrid webinar/focus group approach will be an effective engagement strategy to obtain information regarding therapy access, for the highly sensitive population of caregivers of CSA survivors.

## Materials and methods

### Approach

We hosted two hybrid webinar/focus groups to increase participant engagement in a workshop style format using the webinar function on Zoom (https://zoom.us/). This webinar function allows use of the Q&A function to respond anonymously to the moderators. The option to “up-vote” popular questions was considered useful for keeping track of questions during sessions. During the one hour hybrid webinar/focus group session, the main moderator (a graduate student research team member) described research and programming at Little Warriors for about 10–15 minutes. After this, the moderator then posed five open-ended prompts (one at a time) to stimulate audience feedback for the remainder of the one hour session. The main purpose was to investigate how a multimodal treatment program was received for caregivers of CSA survivors. Feedback discussion was facilitated by the main moderator and two staff members, and the prompts were partially adapted from the Alberta Quality Matrix for Health [16]. From a trauma-informed perspective, this supported a ‘warm-handoff’ between someone more familiar to the caregiver(s) (i.e., the staff) and the researcher. Prompts were comprised of:

1. What has been working for you and your child?
2. What hasn’t been working for you and your child?
3. What can we do to improve?
4. How was the intake process for you? Easy? Difficult?
5. What did you/your child find most helpful in the time at Little Warriors?

### Study context

Little Warriors is a novel intensive multimodal not-for-profit treatment program for children and adolescents with a CSA history [12]. The Little Warriors *Be Brave Ranch* (BBR) facility is “camp-like” in order to create a fun and recreational environment for activities. BBR programming includes treatment rounds for cohorts aged 8–12 years old as well as 13–16 year old adolescents. Each individual cohort consists of approximately 6–12 children.

### Data collection

This study was approved by the University of Alberta Human Research Ethics Committee (Ethics review number: XXX). In fall of 2021, all 89 caregivers were invited by Little Warriors via email to participate in a hybrid webinar/focus group that included an open Q&A period. We took everybody we could with an inclusive list of caregivers who were on record with children in this program, thereby representing 89 caregivers and minimizing recruitment bias. For the present study, all caregivers that had a child that was either in or had completed treatment were included. Caregivers were excluded if their child had not participated in the Little Warriors treatment programming. Implied Consent was obtained at the beginning of the hybrid webinar/focus group session, meaning those who participate are indicating they are comfortable with having their responses recorded and anonymously analyzed. For the caregiver data obtained from the hybrid webinar-focus group, we originally proposed obtaining written consent from caregivers prior to the study. However, we found that anonymity was better maintained by the setup of our webinar where individuals were invited to attend. The webinar was a safe environment because personal data was never at risk. Due to the sensitive nature of the population, implied consent was deemed the most efficient method to get information and the ethics committee was satisfied with our proposal. We also did not require follow up so implied consent was adequate. Furthermore, since this data collection did not involve the collection or use of health information, the Research Ethics Committee deemed that there was little risk to participants and that implied consent was both practical and appropriate and better protected the confidentiality of participants—confidential responses from the question-and-answer session were analyzed for attitudes/opinions of the parents/guardians. Participants were also provided with information prior to the group activity in order to make a fully informed decision regarding their contributions.

During the hybrid webinar/focus group, anonymized responses of caregiver attitudes and opinions were captured using the Q&A function on Zoom webinars, so anonymity of participants was protected. All moderators present signed a confidentiality agreement through the University of Alberta’s Human Research Ethics Board. In order to maintain a continuous flow in conversation throughout, the moderator posed questions to the staff for their opinions during times when caregiver responses were not being entertained. The sessions were conducted in English, via Zoom, and lasted approximately one hour. Two sessions were held in November 2021 and both sessions were recorded. Encrypted Zoom links were sent out by Little Warriors and recordings were stored in a secured online file only accessible by research team members.

As a note, three moderators were actively involved in the hybrid webinar/focus group discussions, as they facilitated the conversation and asked questions (other two moderators were present solely for technical support). In this sense, we qualify them as being participants in the study. As a result, their inclusion in the count of participants reflects their contribution to the data collection process. Additionally, we want to highlight that moderators were not passive observers, but rather played a critical role in ensuring the success of our novel approach by engaging participants and promoting a rich/continuous discussion throughout the session. Moderators presented information according to standard script and they brought questions, raised by participants, into the conversation in a nondirective way—participant feedback drove the discussion. The facilitators had no vested interested in the content arising from participants and their job was to be sensitive and receptive.

### Data analysis

We used qualitative thematic analysis to better understand the attitudes/opinions of caregivers with children enrolled in treatment at Little Warriors [17]. The recorded sessions were transcribed (S1 and S2 Files), and two independent coders thematically analyzed the transcripts using Braun and Clarke’s methodology [18]. Qualitative data analysis was used to identify, analyze, and summarize themes in detail. Initial coding of transcripts was completed by two independent team members (XXX and XXX) using an inductive approach. This allowed for inter-rater reliability and confirmability [19, 20]. Steps taken to complete the analysis included the following. First, the two independent team members thoroughly read the transcripts multiple times to immerse themselves in the data. This allowed the team members to gain a perspective of the data without even beginning to code. Once completed, the two independent reviews then began the preliminary coding process. This consisted of the two members creating a Word document that contained a table with the transcript in one column and the open invivo codes in a secondary column. Inductive coding was used at this point in order to ensure that participant voices were heard without the influence of the study question. Second, once created, these inductive open codes were combined and the two team members began searching for preliminary themes (i.e., how the data could be grouped together in a way that made sense) a larger team (XXX, XXX, XXX) was engaged in an additional round of analysis on deductive coding guided by the study objectives. These deductive codes were then combined with the inductive codes, and comparisons occurred if the deductive codes could be matched with the preliminary themes established for the inductive codes which did occur. Third, the themes were reviewed in light of the original data to determine if the interpretation seemed to capture the essence of participants’ words, phrases, and potential meanings. Regular meetings involved discussion, code verification, resolution of discrepancies, and confirmation of final themes with research team members who were bound by confidentiality. An audit trail of memos and discussion minutes was used to verify decisions about themes and to maintain credibility as well as rigor throughout the thematic analysis [21]. Standard qualitative research reporting requirements were followed [22].

## Results

### Quantitative descriptive analysis

89 caregivers were invited and 11 (12%) attended the webinar, which represents 55% of the 20 caregivers who registered. A total of 16 unique participants, including caregivers, clinicians and moderators, contributed to the data in the present thematic analysis (See Table 1 for breakdown). Hybrid webinar/focus groups were conducted on November 22, 2021 (Session 1) and November 25, 2021 (Session 2). Two caregivers intended to participate in Session 2 but could not join due to limited time or technical difficulties. A research team member followed up via email twice to provide additional opportunities for feedback, but no responses were received. This preliminary study is part of an ongoing program evaluation, so data saturation will be determined in future qualitative evaluation studies [23].

**Table 1:** Participants (N = 20) that attended the hybrid webinar/focus groups.

Hybrid webinar/focus groups	Caregivers	Moderators	Staff
Session 1	5	2	2
Session 2	6	3	2

Note. Two of the moderators and two of the staff members participated in both Session 1 and Session 2.

### Thematic analysis

The hybrid webinar/focus groups conducted yielded several major themes. Themes include: (1) Challenges of starting and maintaining treatment, (2) Therapeutic benefits of specialized treatment, and (3) Barriers and facilitators of treatment. These major themes and related sub-themes are summarized in Table 2 and are presented with greater detail in the following sections.

**Table 2:** Supporting quotations from thematic analysis.

Themes	Sub-themes	Generated Codes	Supporting Quotations
**Challenges of starting and maintaining treatment**	**Emotional burden of intake day**	Something about the emotional impact of intake day (temporary holding code “emotional burden of intake day”)	“[I]t [intake day] can be quite an emotional day as well as an exhausting day for our caregivers.” (Staff, W1). “[T]he day [intake day] I had to leave him was very hard.” (Caregiver, W2). “[G]ot a call back right away. The interview[er] was kind and empathetic, it wasn’t rushed. And I felt listened to and I’m very appreciative…” (Caregiver, W2) “[E]motional, but at the same time, felt relief there was help out there.” (Caregiver, W1).
	**Staying in treatment**	Something about practical barriers to treatment enrollment as well as treatment outcomes (temporary holding code “treatment barriers”)	“[L]ots of homesickness” (Staff, W1) [D]istance can be a factor.” (Staff, W1). “He [child in treatment] also called me every day for the first three weeks begging me to pick him up.” (Caregiver, W2). “[H]er peers are not familiar with hearing or seeing [the concept of voicing boundaries].” (Caregiver, W1).
**Therapeutic benefits of specialized treatment**	**Not feeling alone**	Something about connectedness and feeling alone (temporary holding code “high cost of isolation”)	“[S]o that they know that they’re not alone, they’re able to have those connections, and hopefully build some sustaining peer support.” (Caregiver, W2). “Our girl has been super lonely most times here at home. But she is now in school making a couple friends. So she is really looking forward to her socialization with her schoolmates.” (Caregiver, W2).
	**Feeling safe and supported**	Something about what was helpful at Little Warriors programming (temporary holding code “therapeutic benefits”)	“[T]eens have a lot of time to really build that rapport relationship with us and […] are able to slowly open up kind of out their own on their own time, which is something that isn’t so available with other clinics…” (Staff, W1). “[T]hat’s one piece I know through COVID [t]hat was a little tougher, just because we’re […] limiting visitors as much as possible. But we do have that virtual tour online, which is really helpful…” (Staff, W1). “We can use a lot of the same language because the program helps even really young children talk about more mature concepts such as boundaries, mindfulness, self care.” (Caregivers, W1). “So the smaller group setting was great to make her feel comfortable” (Caregivers, W2).
	**Moral injury wounds addressed**	Something about moral injury/PTSD-related factors addressed in treatment (temporary holding code “high cost of psychological wounds”)	“But what really worked was strategies he was taught at the ranch, they totally took the shame out of his pain. As he moved forward, this was one of the most important things.” (Caregivers, W2). “And it [incident of child sexual abuse] doesn’t define them.” (Caregivers, W1).
	**Trauma informed staff**	Something about staff being emotionally attuned, present, skilled, and empathic (temporary holding code “supportive staff”)	“[W]e’re always getting trained in different types of trainings. So trauma, trauma one on one, I think just the structuring of the program itself is really trauma informed…” (Staff, W2). “[O]ur staff sanctuary. So that’s where the staff can go to really just ground themselves in between breaks or wherever they might have some […] time to do that. In addition to those debriefs, […] I think most of the participants know this, but every fifth week, we actually don’t have any children or youth on site.” (Staff, W2). “[T]he staff, they were kind, responsive, observant, understanding and still firm and what they expected from my child. programs are only as good as the staff that run it…top notch therapy, caring and knowledgeable staff, again, on the clinical staff side.” (Caregivers, W2).
**Barriers and facilitators of treatment**	**Future areas to scale up**	Something about areas of improvement for Little Warriors (temporary holding code “future clinic directions”)	“And it’s […] difficult to find some of those resources and even posts, post treatment or post program. There’s still areas where this individual is acting out.” (Caregiver, W1). “[We want] Government funding for the ranch.” (Caregiver, W2). “. . .[A] school counselor can help be a mending piece between a client or like a kiddo. So I found what generally when our kids are connected with a school counselor, it appears like they’re more at ease with school, and they generally feel a lot more connected to their school.” (Staff, W1). Yes, I was super excited to hear about Indigenous elder coming to the camp. But I was sad, it could not happen with COVID. Yeah, so hopefully, things can open up again and have that inclusion because it does really sound valuable.” (Caregiver, W2).
	**Residual psychological wounds**	Something about what has not been working for the child (temporary holding code “persisting psychological/interpersonal difficulties”)	“[W]hen we work through trauma, and in kind of going through that process, it’s, it can be kind of a roller coaster, right. And a lot of the times once people start kind of getting into some of that therapeutic work, it can look like a digression.” (Staff, W1). “[O]ur daughter wanted to go home. That was really hard, but she ended up working through it and stayed.” (Caregivers, W2). “[W]hen there are times of her daughter calling home crying about when there was a disagreement with another cohort that they’re able to work through. And the following day…they would call and say that everything was fine. She was okay to stay [at the treatment facility].” (Caregivers, W2).
	**Challenges of maintaining self-care**	Something about self-care and associated financial barriers (temporary holding code “barriers to self-care”)	“We have kids that think self care can only be bubble baths. And so teaching them like no, like, there’s things that you can do outside of that for self care and getting them to internally reflect and learn how to take care of themselves, really, without relying on outside things.” (Staff, W1). “Like we always say…cost should never affect your self care.” (Staff, W1).

#### 1. Challenges of starting and maintaining treatment

Caregivers and staff identified the challenge of keeping the children in treatment rounds until completion. These challenges are not surprising given the intensity of the childrens’ trauma histories and the substantial commitment on the part of the children to go to an unfamiliar place for several weeks away from family. Sub-themes include: (1) Emotional burden of intake day and (2) Staying in treatment.

*1*.*1 Emotional burden of intake day*. Intake day was unanimously identified as an emotional day for both caregivers and staff. For staff, they have gone through the intake process for many families and identified common themes of the experience being difficult emotionally. For caregivers, there seems to be an emotional toll that is accrued during intake day. Caregivers pointed out the difficulty in saying goodbye to their child at the facility. However, caregivers also mentioned that the burden of intake day was well worth it, given how it helped their families and the fact that the interview process was not rushed. One caregiver was relieved that there was in fact treatment available for children with a history of CSA. The fact that this multimodal treatment programming exists seems to offer families hope that their child can work toward recovery even after severe early life trauma.

*1*.*2 Staying in treatment*. Given the high degree of heterogeneity in the Little Warriors’ population, some families found that maintaining treatment was hard. These factors can be internal such as homesickness and also external such as location. Living away from their families in a new environment seems to be a difficulty that many children face during the first few weeks of treatment. However, challenges may also reflect mental health difficulties. Another factor included school, where missing out on substantial schooling means falling behind peers in terms of grade level as well as emotional maturity.

#### 2. Therapeutic benefits of specialized treatment

Aside from the challenges reported by caregivers and staff, there were several therapeutic benefits observed. In this theme, both caregivers and staff identified specific areas that provided therapeutic benefits for children at Little Warriors. Little Warriors splits children into small cohorts and uses a multimodal approach with several different types of therapies used throughout treatment. Given the nature of this multimodal programming, the benefits described make sense, as children have the space to gravitate toward specific treatments that they find most helpful. Sub-themes include: (1) Not feeling alone, (2) Feeling safe and supported, (3) Moral injury wounds addressed, and (4) Trauma informed staff.

*2*.*1 Not feeling alone*. Child sexual abuse can be a severely isolating experience not only for children but also for families. The high cost of feeling isolated is associated with a response to trauma, where children believe that they are the only one affected by abuse. As children come to Little Warriors and start interacting with their cohort, they realize that they are in fact not alone. It seems that the cohort approach helps to mitigate some of the harmful effects of CSA.

*2*.*2 Feeling safe and supported*. One key factor that both groups identified to achieving optimal treatment outcomes is feeling safe. Clinicians emphasize giving ample time to adjust and build rapport in efforts to support children. Having the resources to create a safe environment where children can gradually open up emotionally appears important, as the treatment commitment can be frightening and overwhelming to families. Ensuring caregivers have space to ask questions is important. Staff ensure that families are fully informed of the supportive environment by adapting tours to the COVID-19 virtual environment. Programming also plays a role in facilitating psychoeducation. Psychoeducation then allows children to bring these skills back home and to continue their relationship with their caregivers post-treatment. The use of small cohorts was also instrumental for creating a sense of safety and comfort for children.

*2*.*3 Moral injury wounds addressed*. Children with a history of multiple and prolonged incidents of abuse have enormous stress in their lives and coming to an unknown treatment facility with strangers can be an added burden. Thus, the road to recovery is a gradual process. One topic that arose across caregivers was the devastating impact of shame associated with CSA that was adequately addressed in treatment. It seems that the multimodal treatment approach was effective in addressing issues related to the construct of moral injury in terms of shame specifically. The careful attention to addressing psychological wounds at Little Warriors helps children to start to build resilience.

*2*.*4 Trauma informed staff*. The staff at Little Warriors are trauma informed (as demonstrated through ongoing training as well as clinical supervision and programming expectations) and they have specialized training (Therapeutic Crisis Intervention, through Cornell University) to manage behavioral and/or emotional crises. The staff (are supported through management) and value their own mental health as well which serves as great models for the children they interact with on-site. It seems that having rest/compassion fatigue weeks with no treatment programming (and no children or youth on-site) is important to maintaining the wellbeing of staff and to avoid burnout. One of the key principles of trauma-informed care is to foster empowerment in patients, which in turn sets the stage for establishing trustworthiness.

#### 3. Barriers and facilitators of treatment

In light of the therapeutic benefits mentioned above, caregivers and staff reported examples of factors that hindered as well as facilitated treatment progress. In this theme, both caregivers and staff identified specific factors that got in the way of the treatment for their child as well as factors that need to be scaled up in the future. Factors identified in this theme related to the children’s wellbeing, resources at Little Warriors, and staff. Sub-themes include: (1) Future areas to scale up, (2) Residual psychological wounds, and (3) Challenges of maintaining selfcare.

*3*.*1 Future areas to scale up*. There were some areas that caregivers identified as needing scaling up. In terms of resources, this may be addressed by re-allocating the existing resources to better support families post-treatment, or potentially adding additional resources. Ensuring that schools have counselors can be of benefit to children as they make the transition back to school post-therapy. Specifically related to COVID-19, there were some programs that were postponed due to public health measures, but future directions could include ways to adapt to the virtual environment. Also, it seems that cultural activities are valued and received well by families and caregivers at Little Warriors.

*3*.*2 Residual psychological wounds*. Due to the severity of trauma in CSA, there can often be residual psychological wounds that factor into how well a child progresses. It seems that treatment progress is not linear, and this is not surprising given the severity and nature of trauma these children experience. Some children may have persisting difficulties with peers. Others want to leave treatment early to go back home. In light of these interpersonal challenges, sometimes persevering through them with the support of trauma-informed staff and supportive family is the best course of action to facilitate recovery.

*3*.*3 Challenges of maintaining self-care*. Staff spend time educating what self-care is to children by clearing up misconceptions—staff also model self-care themselves. Staff self-care skills play an important role in protecting against things such as burnout and vicarious trauma. Moreover, through educating and modelling, staff communicate to children and families the non-negotiable nature of maintaining self-care as it relates to emotional regulation and wellness in general.

## Discussion

This study demonstrated some of the unique challenges that caregivers of CSA survivors face in relation to therapy access. Due to the difficulties of eliciting feedback from vulnerable populations, this study adopted a unique hybrid webinar/focus group approach to engage a sample of caregivers of CSA survivors at Little Warriors. We found that piloting this novel approach was an ethical and efficient way to stimulate engagement in a highly sensitive clinical population that has been historically difficult to engage with at our facility. The use of this hybrid webinar/focus group strategy may be applicable to other contexts outside of health research due to its flexible and efficient infrastructure using video conferencing technology. Importantly, this approach is also adaptable to the COVID-19 virtual environment and has shown encouraging results in a sensitive population for the present study. At the highest level of scale-up, this approach could be used in a similar fashion to consensus conferences [24]. We discuss barriers and facilitators that arose from our thematic analysis and also several advantages of our hybrid webinar/focus group approach below. Findings may not be generalizable to all caregivers of CSA survivors though since this study was conducted with a sample from a specific facility.

One of the core principles of trauma-informed care is to build a strong therapeutic alliance with the client [25]. Opportunities to start developing rapport begin with intake day when caregivers hand off their children to facility staff. Both caregivers and staff reported that intake day was emotionally burdensome in our thematic analysis. Given the theme reported in, “Emotional burden of intake day”, it is important to note that avoidance, by both the caregiver and child, has been previously found to predict child outcomes as well as treatment drop-out [26, 27]. In order to bolster a therapeutic alliance in a trauma-informed approach and to improve treatment retention, clinicians can play a role in supporting caregivers to actively engage during the intake process. This includes making sure caregivers are well informed as staff discuss and set expectations for therapy, outlining treatment topics that will be covered during the course of care [28]. Staff can also ensure adequate time to address caregiver concerns and questions early on in treatment. Finally, there should be resources available for emotional support if caregivers decline treatment (or for post-treatment) in the form of psychoeducation.

Barriers to engagement/recruitment previously found in the literature relate to the time commitment and value for participants [13]. The present hybrid webinar/focus group approach was able to address this barrier, in a cost-effective and efficient manner, by having a Q&A session where caregivers could obtain any clinical programming information they were looking for directly from staff at Little Warriors in 30–60 minute sessions. Caregivers also learned about the value of future research studies planned at Little Warriors, so that they have the option to participate in the ongoing program evaluation for children and adolescents’ recovery. We believe that the success in these responses was a consequence of creating a safe environment wherein both stakeholders were incorporated (i.e., the clinical staff and caregivers) and having a continuous conversation sustained throughout the whole session [29].

Sensitive populations can be especially challenging to engage with due to stigma and the need for establishing trust [7]—populations such as CSA survivors and their families are one example. For health research in general, obtaining information about patient identified priorities and areas of improvement are essential for improving treatment outcomes and supporting families in their recovery [30]. However, it can be difficult to obtain sensitive information especially in a traditional in-person focus group setting, since disclosure could result in feeling stigmatized [7]. Participants may recognize each other in a traditional in-person focus group setting which may reduce the quality of content shared, especially given the sensitive nature of topics discussed. In terms of individual interviews, participants could feel less inclined to share information in a one-on-one setting if the interviewer is of another gender or cultural background, for example [31], potentially due toa power dynamic affects the quality of information shared. The uniqueness of the present hybrid webinar/focus group format allows for caregivers to give anonymized feedback by typing in their responses in a Q&A chat box, relating to key areas of program improvement and implementation. We carefully considered options for interacting with this vulnerable community and after considerable discussion from an ethical perspective, we decided that maximizing privacy and minimizing contact was the optimal approach to make the context safe and to reduce the possibility of increasing any trauma experiences. This overriding concern for the wellbeing of participants made the anonymized webinar approach a definite choice of advantage—this was also appreciated by the ethics committee. This anonymized approach to engagement is distinct amongst traditional methods of data collection [32]. Given the unique challenges of working in a highly sensitive population as well as the elevated number of no-shows reported in previous literature on virtual events versus in-person [33], we still generated a sample within the recommended focus group size in the COVID-19 virtual environment [34, 35]. Interestingly, the clinical director of Little Warriors noted that some historically difficult-to-engage caregivers ended up participating in the present hybrid webinar/focus groups. We suspect that this is a result of using anonymized responses in a video conferencing format, which facilitated open communication about sensitive information. Furthermore, protecting the anonymity of participants has been recognized as a factor in establishing trust, which is crucial when engaging with sensitive populations [8, 9].

Several limitations to this novel recruitment strategy approach include: first, the hybrid webinar/focus group approach has not been evaluated in other contexts, but is flexible enough to be useful with other sensitive/vulnerable populations. Anonymous responses captured through video conferencing allowed participants to identify patient-identified priorities and information regarding treatment in private.

Second, content discussed throughout the hybrid webinar/focus groups could be potentially triggering to participants. The University Ethics Board accepted our rationale that obtaining implied consent at the beginning of the session represented a safety measure for participants in addition to provision of contact information for research team members and Little Warriors to direct participants to appropriate care pathways, if needed.

Third, access issues posed a limitation specifically for participants who missed the hybrid webinar/focus group. We did our best to ensure two different evening times were scheduled for sessions to allow for caregivers to attend after work hours. Additionally, we provided information about downloading and using the video conferencing platform—a technical moderator was also on call to troubleshoot any user difficulties. Despite these measures, two caregivers who indicated interest in attending the hybrid webinar/focus group session were unable to join due to availability [i.e., scheduling conflict arose unexpectedly] or technical difficulties [i.e., video conferencing platform was not working for the participant]. One can always expect subject loss in internet-based environments because quality of access to internet can vary. In studies of this kind, it may be possible to offer internet access at a specific site in the future to offset technical difficulties (i.e., offering an alternative internet access location at a secure hospital site).

Fourth, overall response rate was low compared to the number of caregivers originally invited, but 55% of registrants attended the seminar [far in excess of our expectations for a successful outcome and around the level of registrant attendance for general business webinars [15]]. Those participants who attended may have differed from those who did not, which presents a possibility of a self-selection bias and reduction in generalizability.

## Conclusion

Our thematic analysis shows that clinical staff can collaborate with families to actively foster a strong therapeutic alliance. Caregivers identified that resources could be better allocated to support families post-treatment. This hybrid webinar/focus group approach efficiently and ethically engaged caregivers of CSA survivors with the benefit of informational value. Invitations were sent and individuals voluntarily registered without follow-up calls or the need for individual person to person interaction. We acknowledge that our study focused recruitment on a specific caregiver population with children enrolled at Little Warriors. Little Warriors research team members confirmed that caregiver participation has always been very low at Little Warriors. Despite this history, we gained a rich qualitative dataset from 11 different caregivers using this approach, within the COVID-19 context. Our sample was also within recommended focus group sizes of six to twelve, so these encouraging preliminary results call for further evaluation [35]. This approach also has an added benefit in that it is completely virtual and adaptable to the COVID-19 virtual environment. Although this pilot study and approach was successful in obtaining feedback at our facility (i.e., obtaining a rich qualitative dataset), further efforts to replicate this preliminary approach in other populations (i.e., substance misuse, severe mental illnesses, and youth with other early life trauma) outside our clinical context are recommended.

## Supporting information

S1 File Full anonymized transcript of webinar session 1. (DOCX)

S2 File Full anonymized transcript of webinar session 2. (DOCX)
