# Supplementary material for: Access to therapy for child sexual abuse survivors: Preliminary dialogue of barriers and facilitators between caregivers
Source: PLoS One. 2023 Nov 17;18(11):e0294686. doi: 10.1371/journal.pone.0294686 (PMC10655970; doi:10.1371/journal.pone.0294686)
Supplement: S1 File — (DOCX) [file pone.0294686.s001.docx]

LW webinar 1

Mon, 11/29 1:10PM • 55:38

**Moderator 1** 00:24

However we're just going to get started a couple minutes. Just wait just for a little bit as people start coming on slowly thanks for your patience. Did you say you guys sent out a reminder for the link today or was just done the first time?

**Moderator 2**

I believe XXX so wonderfully sent out a reminder about it

**Moderator 1**

yeah perfect okay, then I think I'm gonna just get started slowly if people are still joining, I can accept them in as they come in. It looks like we have three in the audience. So thanks for joining everyone. It's great to be able to engage with you in this virtual way. So this is an information webinar in the Little Warriors context. I'll just start by introducing myself. It's really nice to meet you. It's I'm XXX, and I am a Master's of Science students at the University of Alberta Department of Psychiatry and my main project, it's working in the in the Little Warriors context. So I've been working with many staff members there for the last. It's been about 10 months or so. And I'm really excited to just be able to engage with you all today. Joining me are some of my colleagues, XXX, she's going to be able to help out just in that technical capacity. So thanks, again for joining. And then I think you will have had some kind of contact with either XXX or XXX. They are the XXX. So yeah, XXX and XXX, thanks again for for joining. And yeah, look forward to chatting more. So. Yeah. Before I hopefully you can see the slides. XXX, can I just get a thumbs up? Okay. Sounds good. Yeah, before we get started. Yeah, just I'm just required to go over a very brief slide, based on what informed consent is. So by engaging in this webinar, your consent to participate, it's implied. That means you're under no obligation to participate. You can refuse to answer any questions you don't want to answer or ones that you're uncomfortable answering. session will be recorded for the purpose of analyzing the responses afterward and they'll be all anonymized. your identifying information and any other identifiers actually, they'll only be available to to the research team, and they will never be disclosed to the public. So, today, the benefits of attending the webinar, it's, it's to help you hopefully gain a better understanding of future research projects and how we can better support you and your child. Ultimately, in terms of risks, there actually are no significant risks. But you know, some things that you think of some things that that you may contribute to the discussion, it might be emotionally triggering. So if that if that's the case, feel free to reach out to me or to Little Warriors at any time, we'd be happy to point you to the appropriate community context, and care pathways in that case. I'm also required to just briefly show this number, if you have any questions regarding your rights as a participants, specifically, here's the number for the research ethics office. But again, if you just want a more direct contact, feel free to reach out to me at any time, I can provide my number and contact information at the at the end of the webinar. Okay, so yeah, thanks. Thanks, again, for being here. What are we talking about today? Well, I'm going to talk just very briefly about the Little Warriors programming. I'm sure most of you are familiar with it. But just just to set the context and the stage, just review some of those that might be a nice refresher for some of you. And then I'm going to talk about some of the relevance of the clinical measures being taken and captured a Little Warriors. And the the main part of the webinar, it's really opening up the floor, because we're really interested in hearing your feedback. Yeah, we just to protect your privacy, though, we just asked you to use the chat function, specifically, the Q&A. It's, it's very well laid out in this webinar function. So feel free to use that, that function at any point. And after I'm done talking just a couple minutes, I'm going to go through a couple specific questions that I'm curious to hear your feedback about. And that time, just Yeah, feel free, feel free to use that to provide your feedback. And we'll be can have discussion there. So Little Warriors, the Be Brave Ranch, this is the the treatment program. And it's, yeah, when I start reading about it, I It's really quite the program because they, they use that multimodal approach to therapy. And I really like that in terms of offering different things such as cognitive behavioral therapy, you know, EMDR, yoga, art therapy, animal assisted therapy, there's just so many. But yeah, I read that many of the children, they receive over 200 hours of direct therapy, which is so great to hear that those kind of resources are put in place. And yeah, there's a children's program and the teens program. Outside of that be brave, ranch context, there's also the Be Brave Bridge, online programming, you may or may not have heard of this yet, but I've actually gone through this, myself in full and it's just a series of online modules, which you can complete at your own pace. And they go through very important and key concepts such as the importance of the quality of the relationship between parent guardian child, or, you know, things like being aware of toxic stressors, just importance of the family environment, etc. So, if you if you, if you haven't gotten the chance to look at that, I would highly encourage it, it's, it's been very helpful for, for my own understanding as well. Um, so in terms of the future research studies that we're thinking about and planning for, for the near future, we really want to do something that is called mixed methods. So what that means is, we want to be able to collect both qualitative data from interviews and focus groups, where we ask questions, and we're able to hear from you all more an extended way. But also we want to continue to be able to do the quantitative data from the clinical surveys, which I think most of you will have done at some point you have some kind of experience with with those, whether it be on paper or just online as well. So those are the kind of things that we're hoping to to scale up in the future to continue to learn more about the program learn more about individuals and treatment to ultimately continue improving the program. So yeah, we really just want to be able to open up the floor and hear from you. And the reason why is because because we want to gain a fuller picture and a fuller understanding of the types of strengths, the types of gaps and areas where we can improve in terms of the treatment context, at Little Warriors. But to do that, you know, we really value your opinion, because you've either gone through this program or perhaps you're going through this program currently. And yeah, your your opinions and your feedback, it's it's very valuable, because we were able to take that we're able to discuss as a research and clinical team, and then ultimately be able to tell that to policymakers, to people who are continuing to innovate treatments for ultimately, for the benefit of children, adolescents, enrolled in Little Warriors. So that's kind of that's kind of the context. And basically, right now, how this is going to work is, I'm just going to go through a couple open ended prompts or questions. I'll just just go one at a time. But feel free to to start thinking about it. Feel free to start giving some feedback in the Q&A function, you can be long answer short answers. They're really all valuable. And I really do mean that. But as you're thinking of these things, maybe I'll just open up for first to XXX or XXX, just from a clinical standpoint, from your positions, as, you know, clinical transitions coordinators, would you be able to potentially speak to this question very briefly, like what has been working for you and your child? And it's, you know, it's a very general prompt, but just based on your conversations with child and adolescents, maybe you've had, I'm sure you've had discussions with with many families, like type things that have often come up themes approaches where, you know, the caregiver child relationship, it's, it's been, it's worked, it's been very beneficial. So I'm just I'm just curious to know.

**Moderator 2** 12:05

So I think we have a lot of conversations with the families in terms of especially what the kids learn at the Be Brave ranch and bringing it back home, to the families. And I know that can be a big learning curve. For a lot of the families, some of this languaging that we're using at the Be Brave Ranch is something totally different than what they might be using in the home or, if at all. So there's, we always say that the kids are only one piece of the treatment rate. And we always want to make sure that the families are understanding of what's going on at home too. I'm trying to think if there's anything else I don't know, XXX is something else kind of popping into your your head that you can think of?

**Moderator 3** 13:00

Yeah, I think just even considering, like thinking about what has worked for you and your child, even in the sense of like how we've communicated potentially, where we have provided treatment that you've seen work with you and your child. And just think of those kind of details that kind of stick out to you of what has were, what you what you're bringing potentially home, kind of as XXX said, what you're utilizing in your day to days now. And things like that?

**Moderator 1** 13:44

Do you find that most of those perspectives, they come from your direct interactions with children lessons? Or is it more from like stories you hear, like on the phone, families recall?

**Moderator 3** 13:58

I would say there's a mixture for me, given that I work with adolescents, I think that they're very are very well generally able to vocalize when things are changing at home kind of what they've noticed change at home. And then guardians are really, really reflective a lot of the time and able to tell us like oh, like I've seen this kind of change. So now I'm able to do this with them. And it's working really well. Or oh, like they're learning the language of boundaries. And now I know what to say when they're breaking my boundaries and things like that. So I think the, for me, at least I see a lot of our teams being able to come back after like in the rounds, two threes and fours able to kind of say like, oh, like these things are changing and working for me and the guardians who are able to do very well.

**Moderator 1** 14:56

Right. It's awesome. Thank you Okay, I just got one question here. I'm just gonna pull it up. Okay, thank you for your comment. I'm just gonna, I'm just gonna read it out. My daughter can verbalize what a feeling is now instead of being angry or not knowing how to tell me what is wrong or even what is good. We can use a lot of the same language because the program helps even really young children talk about more mature concepts such as boundaries, mindfulness, self care. That's, that's really interesting. So like boundaries, mindfulness, self care – XXX, XXX - do you know if those are specific concepts that are talked about, like in the little warriors curriculum?

**Moderator 2** 15:51

Yeah, absolutely. I think I think that's probably an ongoing theme. To be honest, I think they talk about it quite directly in different group therapy sessions and things like that. But I think that constantly, we're always bringing up boundaries and self care. And I think that's what's so, so awesome, just about our therapeutic milieu is that if you, you're kind of more formally talking about it in the group therapy session. And then the kids returned back to kind of like their home, they're in their cabin in the more kind of the personal part of the day, and just having kind of more of those informal interactions with their peers. And those youth workers are the ones that are kind of reminding them of boundaries, and or, Hey, you know, we've had a really tough day, it looks like that maybe we can do like a self care night tonight. So it's, it's kind of over over the span of different areas that the rounds, which I think is kind of cool. So yeah, definitely formally taught about but also informally as well.

**Moderator 1** 16:58

Yeah, really, like, you mentioned, self care. And that's just tying into the comment that was just made, you know, to be able to use the same language. I think that's, that's, that sounds so key, I'm so glad this participant felt that way. You know, even just from an education standpoint, just, you know, make sure we're on the same page. And I wonder, Have you have you kind of seen some differences between, like the children's program, the teen program? Like I think, you know, as you get older, it's, it's perhaps more more natural to be able to talk about these things. But assume in the children's program that might be a bit more difficult, or maybe it just takes more time? I was wondering, do you do you resonate with that? Or do you find it kind of different?

**Moderator 3** 17:50

For me, I would say that it's definitely individual for the child a lot of the time, as well as it depends on kind of how foreign the concept is for them, and how they view that word, especially for self care. We have kids that think self care can only be bubble baths. And so teaching them like no, like, there's things that you can do outside of that for self care and getting them to internally reflect and learn how to take care of themselves, really, without relying on outside things. Like we always say like cost should never affect your self care. Like, you don't need to go and buy bubble bath to take care of yourself, like that's think of a different way to do it. So I think with the teens, you can go a bit deeper into that, just because they're older, and you can really challenge them a bit more to reflect on, like, what can you do to take care of yourself. And that's something we have a lot of graduates, too, that still connect with us. And they're always like, oh, like, my self care just sucks right now. And so we always remind them like, well, what can you do for self care? Like, what can you do today, like in this moment, like tonight, in this week, and we make it a day to day concept, like it's not something you do just when you're low? It's something you'd have to do every day.

**Moderator 2**

I find that XXX, correct me if I'm wrong, but I think with a lot of the kids there, they're really just trying to explore and figure out what actually makes them feel good. Like for a lot of the kids, they haven't actually even taken that time to figure out what makes them feel good. And I think in the children's program, we we do a lot of sensory work to try to help them figure that out. So it kind of matches well with their developmental level. So it's not that they necessarily need to understand why it's so important and kind of more of that cognitive piece of self care but that that you know, If I just like, rub my hands with lotion that feels good, or if I, if I look at these pictures that's visually stimulating, that feels good. And that can be a part of my self care. And I think with the teens that kind of has taken that next level of talking about the importance, kind of more from a philosophical level, like why we need to do self care and and being grownups and being able to know how to prompt ourselves to do self care, because it falls on the wayside of all of us. Right. It's it's hard to maintain that. But I think with the kids, definitely just starting with the basics, for sure

**Moderator 1** 20:35

That's awesome. Yeah, thank you, I heard a lot of great things like I heard, you know, being able to sort of partner with the child, whether it's a staff capacity, or parent guardian, almost kind of a negotiation evens, at some points, to be able to be creative in finding different ways of self care. Yeah. Thank you. Just for sake of time, I'm just gonna move on to the next question. This is kind of related. But this question is more about what hasn't been working for you and your child. And what I'm really getting at for this one is, you know, maybe there's been instances where, or maybe common themes that have come up where you have been frustrated with your child, or times where difficulties arise in the family environment in the house. And yeah, again, maybe I'll just open up to XXX and XXX just briefly, in the Little Warriors context, have there been instances where, you know, certain approaches have been used, or just any kind of staff engagement? Like, it hasn't really worked out? And you kind of took note of them a couple stuff? Like, just wondering if you could speak to that?

**Moderator 2** 21:48

Yeah, I think, a big piece, from what we know of when we work through trauma, and in kind of going through that process, it's, it can be kind of a roller coaster, right. And a lot of the times once people start kind of getting into some of that therapeutic work, it can look like a digression. So we always want to tell the parents that sometimes it is tough once they're going through some therapy and, and sometimes I'll bring up behaviors of eight never seen before. And that's really, really challenging. And sometimes that is brought home with them. And it kind of looks like like things are going really poorly. But that's just a really common. Common, I don't want to use the word side effect, but just kind of a common thing of having to work through all the hard stuff that they're going through, right. And so we always talk about how therapy can kind of look like a roller coaster. And it's not not all pretty all the time. And that's something I definitely sympathize with families when when the kids are going through that for sure.

**Moderator 1** 22:58

Yeah, thanks. Thanks for mentioning that. I'm just going to read out one of the comments, thank you for your feedback. I'm just gonna, I'm not gonna say the name just to protect your privacy, but just this theme of aftercare in instances where individuals for whatever reason, that the treatment was stopped before, you know, the full year was reached. That's, um, yeah, I'm so sorry to hear that. But their, their, you know, their difficulties when that happens. You know, what kind of resources are there on the community? After that? I was wondering, XXX or XXX, have you been able to, I guess, journey or partner with any families? In those instances? Like have you been able to point them to resources? I know on the Little Warriors website, there's there's a lot. But yeah, has that has that come up?

**Moderator 2** 23:52

Yeah, yeah, no, definitely. So just like you said, for like a lot of different reasons. Sometimes we find that the kids aren't quite ready for a program like the Be Brave ranch. So part of XXX and I’s role is to is to help direct them to other resources that we would, we would feel are more appropriate. So that definitely has come up. I don't know XXX. Is there anything coming to mind for you?

**Moderator 3**

I think with that a lot of the times distance can be a factor. So what I found that, like we generally what comes up is we try and connect with somebody within like the more direct community just because then generally they will have a wider range of resources that they know about for that community. Just with right like the limited access to resources to it's always difficult and I think when there is an early decision That's like very hard on families and very hard on the child as well a lot of the time. And so trying to find those supports to kind of put them trying to like surround them with different things is difficult. But that's kind of where we go as we always try and build up their community resources or look within the communities to see if there's anything else out there to better suit the clients, if we're not suited for the clients.

**Moderator 1** 25:34

Yeah, absolutely. Thank you for mentioning that. Looks like we just had two comments. I'm just going to go through these. I'm from the US my daughter had to stop treatment after round two due to severe RAF, I'm assuming that's reactive attachment disorder, correct me if I'm wrong. She is now in residential. I was able to talk with XXX on the characteristics of places I shouldn't be seeking out. This was very helpful. Well, yeah, I'm very glad to hear that. It seems like Yeah, little worries, the kind of keep that community communication line open, which is, which is great to be able to, you know, continue to partner with families even after treatment. But yeah, thank you. It looks like we just had a nother one. Yeah, feel free to jump in. And, by the way. Okay, yeah, I wouldn't say this is something that hasn't worked, but just that my daughter was able to voice boundaries that were almost confusing, or came across as harsh to her peers who hadn't been through such a sophisticated program. It was partly her approach, but maybe also just the fact that she had learned some social skills. And her peers are not familiar with hearing or seeing. Okay. Yeah, very interesting. I wonder, the thing that really sticks out to me, there's just the sophisticated program. That's, I'm wondering if, if that's a theme that is come up in your conversations, XXX and XXX, like, for example, you know, these kids are in school, or they're going to go back to school after treatment. And I assume that, you know, that transition isn't always smooth. And, you know, that's that's probably that can be a source of stress to family members. Or maybe that's something that's directly touched upon from clinicians. I'm wondering if you could maybe just briefly speak to that.

**Moderator 2** 27:45

Sorry, XXX, you just kind of cut out for that. 10 seconds. No, don't apologize. I just for just just wondering if I think what I understood is that if that's kind of been a theme, like with other families, were kind of that transferring of, like, the kind of what the kids learn out to be brave ranch, if that's kind of like an awkward transition back into their community friend group, or is that right?

**Moderator 1**

Right right

**Moderator 2**

Yeah. Yeah, you know, I think I definitely had experiences with that, I think in a different way, I am just recalling some families where the kids get really excited to have learned about what they have learned at the ranch. And, and there's kind of this tendency to overshare. So just kind of on that theme of kind of appropriateness versus inappropriateness, like kind of boundaries and stuff, is something that some of the parents struggle with when the kids come home, just who we need to share the brain for our experience with and when and kind of the appropriateness of that.

**Moderator 1** 28:56

Thank you for providing feedback. Let's see here. I'm just looking at the time. I know we scheduled about 30 minutes, I want to respect your time, but yeah, if any of the moderators need to head out, feel free to I'm happy to stay on as questions still come in, but just wanted to put that out there. Let's see here. Yeah, so another comment here. I'm just gonna not say the names again, just to protect your privacy, but one individual has done good but still not clear about another individual. Sounds like she might have needed a bit more help. And it's it's difficult to find some of those resources and even posts, post treatment or post program. There's still areas where this individual is acting out. I guess that kind of ties into the previous discussion. about resources. Yeah, please feel free for this participant individually just be able to reach out to me or a Little Warriors, we would happily have that ongoing conversation with you. We want to, you know, we want to make sure you have the resources to be able to manage situations you're in. And, okay, another one more. So regarding consent, after round two. I was just wondering if, yeah, has there been instances where there's been some confusion over over consent, like instances where you've had to kind of explain a bit more? You know, I'm just curious to know, from a clinical standpoint, just question.

**Moderator 2** 30:54

Yeah, yeah. So I feel like we teach consent, again, in a very clinical setting, in group therapy, where we actually review, and a lot of times, we of course, have to review the like Alberta laws of consent. And what that actually means and like the ages for consent, what can like the rules around consent, and I know, we go pretty in depth for the adolescent program, just because they can maintain and like handle those conversations. And then it's, again, a continuous conversation that we have in the cabins. And we generally have to repeat ourselves quite a lot around consent as well in round three, and four. Because it is a huge topic, and I think that's a really big piece is, we usually get the repetition of round three and four to keep repeating consent and kind of clear up that confusion. If there is any, of course, and generally, a lot of teens are confused in my, in my, in my students that I've had or conversations that I've had, it's a hard thing to understand. And it's a hard thing to internalize, because they've also had the consent taking away. So it's a weird concept for them sometimes. And then, in the non clinical sense, in the therapeutic milieu we try and teach consent through asking before touching, asking before hugs, asking before like breaking potential personal boundaries and those things too. And again, that's repeated through all rounds. So I definitely could see how there is confusion after a round of treatment, though, because it's kind of like what we do here is we kind of open things up, and then we close things down for at the end of the round. And then we kind of open things up again in round three and kind of close it down. So yeah, I can definitely see where that can happen.

**Moderator 1** 32:57

Right. Yeah. Thanks for giving that example that that's those are very valuable points. I think. I'm just going to move on to the next comments. It looks like one or two people just joined in as well. So in case you missed it, um, yeah, the webinar was just scheduled for about 30 minutes, but I'm happy to stay on and continue to feel questions for up to an hour. But again, if any of the moderators and participants need to leave, by all means, I don't want to keep you too long. But I'm just going to keep going. And yeah, so this one comments. Sounds like daughter has been struggling school fell a bit behind now facing issues where this individual is being made fun of by by teachers, and is now a grade behind. How do I help her and her self esteem in that area? Yeah, that's certainly a big question. I'm really sorry to hear about that. That's, yep, no worries. Yeah. Great to have you. Yeah, I'm wondering, that must be I know, for example, the self esteem there's there's clinical measures specifically taken. But certainly that is an area that, you know, I'm assuming would require ongoing. Follow up. Have you seen instances of that where self esteem has played into the school environments? I'm sure that would be really difficult for some families to navigate. I've there been kind of like tips or things that you've kind of mentioned.

**Moderator 2** 34:50

From what I've seen, kind of be successful, especially because we know with our public program, right? For the children's program, we take them for 28 days at the start, and then a two week periods in between that. So they do miss a lot of schooling to attend our programming. And then without a lesson program, we take them for potentially six weeks out of the year two. So that is a huge weight on our kiddos a lot of the time. And what we have found to be like, what I've at least found to be quite helpful is if they have a school counselor, and that school counselors kind of let in on those issues and those secure like if those insecurities because yeah, like a teacher should never be belittling kind of like a student or making a student kind of feel bad. And a lot of the times a school counselor can help be a mending peace between a client or like a kiddo. And teachers kind of, they were able to kind of bring a bit more light into a situation sometimes. So I found what generally when our kids are connected with a school counselor, it appears like they're more at ease with school, and they generally feel a lot more connected to their school. And they also have a reason to go to school. So I know it's not like a perfect response or anything like it's not a clear cut answer, but it's definitely a resource out there, if the school has a counselor.

**Moderator 1** 36:36

So yeah, I think thanks for that. That's a really important point, I think just that that care pathway in the school, but also just the fact that, you know, there's area for, you know, interdisciplinary collaboration, you know, Little Warriors, schools, that sounds like a really important pathway. So thanks for mentioning that. Looks like we have another comment here. response to the participant who is struggling with miss school and self esteem, one strategy that has worked with my daughter is that I remind her, how important mental health is that she would have a very hard time succeeding in school without being healthy first, I also highly recommend the bridge program for some strategies to talk to your child about the entire experience from abuse through treatment. Yeah, thank you for that comment. That's I think that's, that's really insightful. Very practical. Just I think this is different individual follow up question. So I should speak to the counselor about this and see about having a group conversation. Yeah, I think sounds like based on what XXX said that that definitely might be worth thinking about. And just kind of see, you know, where there are, and I'm sure, you know, Little Warriors would be happy to, you know, help with that process. I know, even just initiating those conversations could could be a source of stress sometimes. So, yeah, thank you for that. Just for sake of time, I'm just going to move on to the next question. This one's more to do with future future steps. So the question is, what can we do to improve? And it's specifically, I'm curious to know, know, if if we were to ask you for feedback that you could provide to the clinical team to future families, even government or funders, to audience members. I'm wondering what, you know, what would you say to them? What kind of feedback would you provide? If you have any, you know, maybe there's, there's a family that is considering joining the treatment program, they're kind of on the fence about it. Is there anything that you would say, and I, you know, I'm sure XXX and XXX, you've, you've definitely had conversations like that. Maybe there's been families that were a little bit hesitant or a little, I don't know, maybe maybe apprehensive and it's certainly a big commitment. I'm wondering, have there been certain things that you have said to previous family members that have helped you know people people understand and increase their knowledge of what what the be brave ranch offers?

**Moderator 3** 39:32

Yeah, so um, something that we're trying to do more often now we just from from kind of my side of things is offering families like a tour of the facility prior to coming. So that can really give them like a good sense of everything before coming in because it is scary, right to go to new place for the first time. So that's, that's one piece I know through COVID That was a little tougher, just because we're We're limiting visitors as much as possible. But we do have that virtual tour online, which is really helpful I find I think a big a big thing was was parents and caregivers worried about being away from their kids for a really long time, lots of homesickness. Oftentimes, ex I think that the parents struggled more than the kids, and I totally get it. Like, it's so so hard. So that was definitely another another big, big piece in my family's.

**Moderator 1** 40:33

Yeah, that's great. Yeah, thank you so much, giving a tour to be able to, you know, be able to communicate to the parents and the family environment, that's, you know, this infrastructure is in place. There's, you know, there have been many chords before. And it's, it's, we're always trying to improve and try to make it welcoming. I really like that. There's just another comment here. One of the biggest concerns going into the program was that it wouldn't be a good thing to be around other kids who have been abused almost as if it would be like a bad influence. But instead, I found that my daughter finally didn't feel alone. She actually said to me that until she went to the ranch, she thought she was the only kid this happened to which never even occurred to me that she wouldn't know other kids had been through this. Wow. Yeah, that's, that's, that's quite common. Thank you for that. And I think that kind of gets at, you know, the, the programming at the ranch where this cohort approach approach is used. Correct me if I'm wrong by I think it's they try to keep it small, like, five, six, maybe seven, for the purpose of connecting, right. And staying, you know, throughout that whole year long treatment? I think that's, yeah, that's, that's great to hear. Thank you. We just have other ones coming here. I'll try to get to everything. So my daughter has had to go to court. And now the accused lawyer has made an application to the court to get to the counseling records from the ranch and the lawyer for us told us that there is a possibility in this happening. I like to be able to have assurance for her that this can't happen. So she can trust that she can speak without fear that the trust of that trust being ripped away again. Yeah. That's that sounds like a very difficult situation. I think it just, it kind of ties in to our discussion before. Like, please don't hesitate to reach out. That sounds like more of more than just, you know, a short, little Chat, I think we'd be happy to assist in any practical way we can, and have that conversation ongoing. So I think I'll leave it there for that one. Thanks for your follow up. That's so true. They do think that they're alone till they go to ranch and see that there's other kids going through the same thing. Yeah, I think, again, that cohort approach, I think it's so important. I'm just going to move on. I think I only have two more questions. So we'll wrap up shortly. But thank you so much for your feedback. So this question is is more specific to the intake process? I think one common may have touched upon very briefly, but I just want to ask him more specifically, how was the intake process for you? Was it easy? Was it was it difficult? Were there certain points where, you know, you may maybe you wish you were informed a bit more, maybe it was really smooth? And again, go just open up the gym? XXX like, I'm sure you've had so many conversations in the intake process. Have there been, you know, instances where it's been difficult, you know, what kind of complaints and concerns have come up? Or maybe the like, more or less, they've been pretty smooth? I'm just I'm just curious to know.

**Moderator 3** 44:06

Yeah, I can speak to that. Really quickly. I do just want to ask the participant that's commenting about court to please directly reach out to XXX and myself just because again, for confidentiality, like we can't reveal who you are. But yeah, please reach out to us if you have questions for this webinar, and we will bring it to our clinical team too, and to have a greater conversation and then to speak about our intake process. This is something we do get generally some feedback on it can be quite an emotional day as well as an exhausting day for our caregivers. It's a lot of paperwork to go through as though some of the feedback weekends as well as a very like timely thing when you have to sign all the paperwork so that is it. Some of the feedback that we get. But generally people do now that we are doing tours, even on intake days and things like that people are very, like, happy to go on the tour. So that's kind of been a positive part of our new intake days now, that COVID is kind of settled down and that they're also able to see the cabins before they leave, like they can actually go to the cabins to say goodbye to their kiddos, where before, we kind of have the kiddo like run outside, but now we are allowing them to kind of walk into the door so they can see the house where their kiddos will be. So I think there's definitely positives and negatives to the intake day. And it. Yeah, it varies in different aspects as well as individually for the parents to

**Moderator 1** 45:49

Yeah, certainly, I could imagine the range of, you know, different reactions and emotions, like it's, I think I know that some people are even at the country, right. So it's, it's really quite the trip and really quite the commitment. Looks like we just have one comment here. It says emotional, but at the same time, felt relief, there was help out there. There, it was emotional to answer the questions for application. Grateful I was directed towards the warriors from our first therapist, or I would have never known about this awesome place. Very grateful. Really glad to hear that. Yeah. And yeah, happy to continue to point you to, you know, the appropriate resources in any way that we can. Um, I am going to go to the final question. Because I know we're reaching the top of the hour soon. So the last question. Let's see your sorry, just one second. What did you or your child find most helpful in the timeout Little Warriors? Um, the reason why I'm curious about this is because I know that generally speaking, I think there's very limited resources out there for children, adolescents who have experienced sexual abuse, not just in Canada, but but broader. And, you know, based on the the small number of clinics that I've seen, there's certainly a broad range of different therapeutic options available. I mentioned it before, but I think this multimodal approach that Little Warriors uses that's very unique. That's very interesting, not only from a clinical perspective, but research. I'm wondering, yeah, I'm wondering XXX and XXX, have you had experience with other clinic clinics with similar patient population? Or maybe just from a staff perspective, based on your clinical team meetings? Have there been certain aspects of the dreamer program that have, you know, proven really beneficial for many people?

**Moderator 2** 48:01

Something that, like, first comes to mind is just that we're a residential treatment programs. So in that, I think that the kids in the teens have a lot of time to really build that rapport relationship with us and, and are able to slowly open up kind of out their own on their own time, which is something that isn't so available with other clinics rate, it's kind of just like that one hour session, like every two weeks, maybe. And that's really tough for the kids to open up. So I really like the fact that we're, we're residential. Oh, XXX, you're just muted still.

**Moderator 1** 48:57

Oh, sorry. No, thanks. I was just reading a one of the comments that they're not alone, and that there's help out there, that they can overcome this. And it doesn't define them. I think. Yeah, those are those are some very big themes there. Thanks for providing that feedback. It looks like another one. The therapy is very immersive, the connection to the cohort that they are, that they are in are allowed. They're allowed to be encouraged to maintain after therapy. Yeah, that's, that's a really good comment right there. Great, thank you so much.

**Moderator 2** 49:39

I think another piece that comes to mind to XXX is, as we spoke to it a little bit at the beginning is the fact that we do have so many different types of therapy and, and we know that kids can can range so so much, right so just because art therapy really kind of kicks it off for kids. That may not be the the modality for Another kid and so they might have that opportunity to really open up and talk therapy right or, or something like that. So I think that's really special.

**Moderator 1** 50:08

Right Yeah. That's, that's pretty interesting. Do you find that? children, adolescents, they kind of gravitate toward a certain type of therapy? Like, does that kind of happen earlier on later on? Or? Maybe it's kind of mixed? I'm just curious.

**Moderator 2** 50:28

Yeah. Yeah. So um, a lot of the times, I definitely see when a team, a lot of them will gravitates initially to, like art therapy and music therapy, a lot of them are just, like, very creative in those senses, or that's how they express themselves. And so they gravitate to that. And then it's like, sometimes it's quite surprising for them, because they're like, oh, like I came in. And I really thought that art therapy would be my favorite, but it's actually art therapy, and group therapy. And so they surprise themselves a lot of the time. In those ways, too. And I think, yeah, that's kind of like, what I've seen is, a lot of the times they'll think they'll one thing will be their favorite. But once they kind of get to try everything, then they'll get to choose a couple favorites that actually worked for them. And you'll see them kind of dragging their feet sometimes for like, mindful movement or yoga, and then like, sprinting towards the music room. So it's great to see.

**Moderator 1** 51:36

Yeah, that's great. Yeah, I think one of the things that comes to mind when I was able to be on site briefly is just the the the collaboration between the therapies, for example, I know that, you know, what the children adolescents do in art therapy, though, I don't think they always do. But they will often discuss that in the individual counseling or group therapy. And I really like that approach, because it allows, or maybe it sort of opens up the door for people to express how they're feeling because, you know, people are able to keep are comfortable with different things. So yeah, I really like that. Thanks for Thanks for sharing that. I'm okay. Yeah, there's nothing. Oh, sir. Looks like there's another comment here. The other part that is so helpful is that these therapists are extremely specialized in sexual abuse, my daughter had been seeing a pediatric therapist before the ranch, and it was just not the same. Even for the parents, the therapist can help you understand a lot more than other specialists who do not understand sexual abuse as much. And yeah, I think that kind of ties in with your comment show like this is a residential treatment program, based on my sort of limited experience, interactions and sounds like the therapists really do have a lot of experience in this specific area, which is I would assume, you know, such a such a benefit to families who are going through this situation. So yeah, really glad to hear that, that the therapy has been received well. Yeah, if there's Yeah, so if there's nothing else, and we're reaching the top of the hour now, I think we're going to leave it at that. But again, I just want to point out, if you have any other questions, comments, I'm always happy and available to to have that ongoing conversation. I'm actually just gonna go to the just one second. Yeah. Feel free to jot down this contact information. That's my, you know, your alberta.ca. And as well, you can always reach out to Little Warriors. There's more information on the website as well, but there's a number right there. Yeah, please, please feel free. I'd love to have that ongoing conversation. I think XXX or XXX, you mentioned that. There's that one question regarding the legal situation. I'm wondering, do you need to provide any other kind of specific contact? Infowars? Do they have your information? I just want to make sure that's addressed?

**Moderator 2** 54:24

Yes, I believe they, they'll have at least my my information from the invite. Okay, but yes, perfect. Yeah. So.

**Moderator 1** 54:37

Yeah. Okay. Great. Thank you so much. Yeah. Yeah, glad. Glad. It was interesting. Thanks for Thanks for coming and engaging. I'm looking forward to doing more things like this. Yeah. For the audience members. Thank you again for your feedback. I think that was really valuable, helpful, and I'm really Excited to be able to further work on this and, you know, just hopefully, ultimately improve the treatment program for the benefit of children adolescents out Little Warriors. And yeah, thanks. Thanks again for joining and if you haven't had dinner, I hope you have a great dinner. So, yeah, I think we'll leave it there. Thanks XXX and XXX. Appreciate it.

**Moderator 2** 55:23

Thank you so much. Bye. Have a good night. Thank you so much. Thank you to the participants for coming.
