# Supplementary material for: Access to therapy for child sexual abuse survivors: Preliminary dialogue of barriers and facilitators between caregivers
Source: PLoS One. 2023 Nov 17;18(11):e0294686. doi: 10.1371/journal.pone.0294686 (PMC10655970; doi:10.1371/journal.pone.0294686)
Supplement: S2 File — (DOCX) [file pone.0294686.s002.docx]

LW webinar 2

Tue, 11/30 11:38AM • 50:49

**Moderator 1** 00:18

In the audience, we're just going to get started a couple minutes. Just wait a little bit to see people are still trickling? XXX, XXX, can you see the PowerPoint slides? Awesome. Okay, well, it is a couple minutes past 630. So I'm going to get started. If people are still joining in, I'll see them in the in the waiting room. But yeah, for now I'll just get started. Thanks for attending everyone. This is informational webinar number two, we had one two days ago on Tuesday went really well. So yeah, if if we got lots of great feedback, and we're able to have a short engagement session virtually. So I'm excited to have another one today, it's going to be the same format. But before I do that, I'll just introduce myself. I'm XXX, and I'm a master's of science student at the University of Alberta. I am in the Department of Psychiatry, been doing my degree for almost 11 months now. So my main thesis project, it's in the Little Warriors context. And just joining me are some of my colleagues XXX and XXX, they're going to be just helping me out as a technical moderator. And then I think most of you will have met, either XXX or XXX. They are XXX out Little Warriors. So yeah, looking forward to the chat. And, more importantly, looking forward to being able to engage with some of you in the audience and hearing some of your feedback. So before, I'm just gonna switch the slide here, before we get into the actual discussion and engaging session, I'm just required to go over this quick implied consent slide. So by engaging in this webinar, your consent to participate, it's it's implied, meaning you're under no obligation to participate. You don't have to answer any questions that you don't want to answer, or ones that you are uncomfortable with. The session will be recorded for the purpose of anonymously analyzing the data afterward. And then your identifying information, and actually any other identifiers, it'll never be disclosed to the public, it's only available to the research team. benefits by attending this webinar, hopefully, you'll gain a better, better understanding rather, a future research studies, and ultimately how we can better support you and your child, specifically the parental guardian or caregiver rule. In terms of risks, there are no significant risks, but some of the things that are discussed or some of the things that you think of might be emotionally triggering. If that's the case, please do not hesitate to reach out to us, me, I'll put up my contact information later or Little Warriors as well. And we'd be happy to point you to the appropriate community resources or care pathway. And I'm just required to quickly show this number right here. If you have any questions or concerns about your rights as a participant specifically, this is the you have a research ethics office number. But again, if you just want more of a direct contact, feel free to reach out to me, I'd be happy to field any questions or concerns you might have. Um, so yeah, thanks again for being here. What are we talking about today? Well, I'm going to briefly go over just some of the little worries programming I'm sure all of you know about it. But maybe it's just to set the context for the discussion today, and maybe just a refresher for you as well. And that I'm going to talk very briefly about some of the relevance of the the clinical measures being taken out Little Warriors. And then the main portion is, we're really interested in hearing your feedback. So to protect your privacy, though, we asked you to use the chat function in the Zoom webinar to provide your feedback. Alternatively, you can actually use the specific q&a function, there's a there's an option to send your comments anonymously. So that's that's kind of the logistics of how it's going to work today. So I'm just going to jump into talking for just a few minutes. Again, Little Warriors Be Brave Ranch, this is the treatment option available for children and adolescents, that Little Warriors. There's a children's program, there's a teens program. And one of the things that stands out to me when I was learning about this treatment facility was the multimodal approach of so many different kinds of therapies and the fact that children, they receive over 200 hours of drug therapy in this camp like facility. And I think another one of the distinguishing factors I learned about was just the cohort approach, individuals are able to be in small groups, six, seven, maybe even smaller, where they're really able to connect with other children, adolescents, similar age, similar experiences, so that they know that they're not alone, they're able to have those connections, and hopefully build some sustaining peer support. So that's kind of the context that Little Warriors works in. Another program that is of relevance here is a debrief bridge online program. I'm not sure if you've heard of it, but it's, it's, it's actually an online set of modules I've personally been through, I really enjoyed going through it as at my own pace. And these modules, they touch upon some key concepts that are relevant in the Little Warriors context, such as you know, how important caregiver child relationship is, things like toxic stress, being able to manage that identify it, being able to know how to identify things related to child sexual abuse, ultimately, for the best treatment outcomes in this context.

So yeah, if you if you haven't been able to take a look, I would highly recommend it. And yeah, if you reach out to us, we'd be happy to point you towards these resources. So some of the future research studies that are being planned a Little Warriors, we want to be able to do an approach where we gain some qualitative information, such as one today, actually, but more so interviews, that whether it be one on one interviews, or focus groups, kind of in smaller groups, where we're able to ask about your feedback. And we're able to have that recorded and again, things like anonymous analysis so that we can ultimately learn more about the treatment program and learn more how we can better support you. And then on the quantitative aspect, more clinical surveys. And I'm sure most of you will have had some some of the experience during measure surveys. But definitely those those are important to capture in order to for the ongoing program evaluation. So yeah, now this is, this is a part where I really want to open up before, we really want to hear from you. And the reason for that is because we want to gain a fuller understanding of the types of strengths, the gaps and the areas where we can improve, both clinically and as a research team. Because the things that we hear from you as participants as parents guardians, we were able to take that and there were ultimately able to communicate that to the policymakers, stakeholders, to continue improving in providing innovative treatments for children, adolescents. So that's, that's really why we, we value your opinion. So at this time, feel free to start using the chat or the q&a anonymous function. I'm just going to go over questions one by one. So we're all on the same page. As things come in, I'll try to direct them as best as possible and we can have a discussion. But the first question really is it's a it's a general open prompts. What has been working for you and your child and some of the things in the first session We talked about just briefly as one participant mentioned that having a chat with the clinical director, a Little Warriors in terms of resources, that was very helpful. Other themes of boundaries of self care, mindfulness, those were things that were very helpful to. Again, this is all relevant to your role as a parent or guardian, as you engage with your child who has had that experience of child sexual abuse. I'm wondering though, maybe, for this time, if we want to touch upon different kinds of attitudes across the treatment rounds, maybe you see differences in the children or adolescents there. Maybe you want to speak to things that have been beneficial approaches, maybe routine routines in your household, like before COVID And during COVID. But as you start to think of those things, and provide your feedback, I'll just start with XXX and XXXl, as well. If you could briefly speak to this. I know that one thing that I was thinking of was I know that there's a regular clinical team meeting. I'm wondering maybe from a clinical staff perspective, I'm sure that's very helpful for debriefing, and even emotionally regulating yourself. And that would hopefully allow you to engage with children out Little Warriors better. I'm wondering if you could maybe touch upon that, or there's maybe other strategies or other meetings that I don't know about that. That would be helpful for the discussion. But yeah, I'd be curious to, to hear a little bit about that.

**Moderator 2** 11:48

Yeah, absolutely. I know that we have we call our staff room, our staff sanctuary. So that's where the staff can go to really just ground themselves in between breaks or wherever they might have some some time to do that. In addition to those debriefs, I think, I think most of the participants know this, but every fifth week, we actually don't have any children or youth on site. So we have all of our staff come together. Those weeks are, are primarily for compassion fatigue, which is kind of like the burnout that a lot of workers in our fields are burdened with. So when we do have those weeks, where we come together, we debrief how the month have gone, if anything personally came up for us, we take time to engage in self care, we have lots and lots of meetings about what had gone on that last month, what we might be able to do better next month, lots of team building, and whatnot. So I don't know if XXX has anything else to add to that. But we're a very close team. And we really value how well we're able to hold ourselves because just like XXX, you said the better we’re, the better place that we're at is where we're best able to serve the kids and youth who come through the program.

**Moderator 1** 13:14

So yeah, thank you for providing that feedback. It looks like we have a couple of comments here. My child doesn't feel alone. She has other peers who get it and understand. And I think that's a theme that really came up last time and I touched upon it with the cohort approach. This is a new comment seeing rugby has been invaluable during one on one sessions. That's yeah, that's so great to hear. I actually don't know too much about XXX, maybe, XXX? Did you want to speak to that a bit? And like what role rugby plays, you know, for the children and all those things?

**Moderator 3** 13:50

Yeah, so XXX is actually considered a co therapist. He is especially trained from dogs with wings. So he was trained to be Little Warriors support animal and XXX being his handler when she's doing one on one sessions with the kids or when she's leading a group therapy session, then XXX, you will join in on those sessions as well. And it's just incredible how you see kids gravitate towards him and how he can be just like he's a living teddy bear. He's so comforting. He's so empathetic, and he's a dog, which is crazy. He's also used in very logical ways to he teaches the kids about boundaries and about respecting XXX space. And, you know, you even have to ask to go into rugby space and how would it feel if somebody was pulling on your ears or something like that?

**Moderator 1** 14:48

Thank you for providing that feedback. Looks like we have another comment here. I'll just read it out. Now. It's been a while since my son was in the program directly. But what really worked was strategies he was taught at the ranch, they totally took the shame out of his pain. As he moved forward, this was one of the most important things. So one on one support and the entire philosophy and culture at the ranch was was important. My hours long chats with therapists, therapists, when I picked him up, was very helpful for me to understand how to navigate the aftermath of the experience. It helped me to gather information about how we should proceed, how to think about challenges and how I could continue to support him. And then just to follow up, your rugby was definitely a huge benefit for my son, too. So great to hear your thank you for providing that feedback. I'm wondering, just before we go to the next question, either XXX or XXX, one of the things that stood out from this comment here was the long chats with therapists from the caregivers side. Do you do you find that you have many follow ups, I guess, either in between rounds, or after final discharge? Is that something that maybe you could just briefly speak to?

**Moderator 3** 16:13

Yes. So on our departure days after each round is kind of completely do our best to schedule in if the guardians are available, a departure meeting. And so that departure meeting is where the therapist can meet with the actual guardian and discuss the progress in person, while they're awaiting kind of our care plan that we sent them after the treatment rounds. So it gives them something right away of what the kiddo has been working on. And then after the programming, it's, we always leave the door open, sometimes we get emails still updating us on how clients are doing a couple years after they've left the program. So yeah, the doors always open for sending update emails and things like that. But during the last care plan, we usually try and do our best to provide resources and such.

**Moderator 1** 17:08

Right, yeah, you mentioned care plan. That sounds like a really important aspect for maintenance. And as you said, keeping the door open. One of the comments, it seems like it's actually related to that the follow up plans, after returning home are concise. Our family physician was very impressed. So that's Yeah, that's very interesting to hear. Another comment here. I was a guardian for my sister, which lives in a different province than me. She was in the teens program, my sister was very reserved. So the smaller group setting was great to make her feel comfortable. Also, the coping tools that we're both giving was very helpful. Yeah, thanks for providing that view. That's, that's great to hear. I think, you know, one of the things I'm hearing from that is feeling comfortable. And I think it's maybe you could speak to this very briefly XXX, XXX. But I know that there's some individuals who come out of province, I think they're even a couple out of country, correct me from wrong. But I mean, that's, that's a, that's a big commitment. So being able to be in a, you know, smaller group where you have your voice heard, I would imagine that that's crucial for finding a place where you feel comfortable sending your children. So yeah, that's, that's great to hear.

18:27

**Moderator 2**

Yeah, so we always try to make the groups a little bit smaller at the beginning, at least for the first, first round in second round, just because in the children's program, at least in the third and fourth round, they tend to actually join up with another group. So when we make the group smaller at the onset of their treatment year, I guess, it allows us to really get to know each each kiddo quite well and just kind of just really build that rapport in that relationship. And then it's always great for them to return with that same group of kids every time they come back. So if one's from Edmonton and other ones from like Australia, that's it's so cool to see them kind of come back and see that familiar face when they come back to the center.

**Moderator 1** 19:14

Absolutely, yeah. So what are here, it's kind of like meeting the child or adolescent kind of where they are kind of easing into it. I really like that. Thank you for providing that feedback. I think for the sake of time, I'm just going to move to the second question. So this is kind of, it's kind of related. And I think we some we may have touched upon small details, but the question is, what hasn't been working for you child? And the reason why I asked this is because, again, this kind of gets that understanding the areas where it's been difficult in the context of having and raising a child with a history of child sexual abuse. Last session we talked about some stuff like falling behind in school, some confusion over the the consent process, some frustrations, being at home too often. I don't think we touched upon this. But I'd be curious to know, when, you know, isolation measures or social distancing measures were put in place. How was that? I'm sure. It's it's very difficult for children not to be able to see their friends in person to have that regular interaction school. And, yeah, maybe as you as you start to think of these things, and provide your feedback. I'd be curious to know from XXX and XXX's perspective. Yeah, yeah. If you have any feedback here? If not, I'll just I can just provide some prompts. Maybe there's been instances from your clinical perspective where kids were homesick or or parents, they really wanted to see their child. So you find that you had to, or you're open to providing that ongoing updates and communication? Yeah, have there been instances like that where it's been challenging, and you've had to make perhaps extra accommodations and just be able to hear what, children analysis episode?

**Moderator 2** 21:23

Yeah, yeah, for sure. So with COVID, we haven't been able to use it as much as we would like. But for those of you who don't know, we have an extra sort of cabin on site that is primarily used for parents and caregivers to stay overnight. On site for the first day or two, sometimes even the whole time, it's it's quite rare that a parent would stay the entire time through a child's treatment. But sometimes it's, it's deemed necessary, whether that be because of the child's significant attachment history or something like that. So So that's definitely a part of what we try to do to make the transition process a little bit easier on our families, with school, and in COVID time, I remember a lot of families talking to me about how, because of the trauma that their children have experienced, they always required pretty specialized supports in school. But obviously, when everything moved to online, that was, that was really tough for them to receive that same support. And so a lot of them fell really far behind, which was super unfortunate, but really common for a lot of our families. I don't know if XXX, you saw the same thing with your teams. But yeah, what I've kind of noticed with our teams is when, during COVID, when that's kind of hit and we have taken kits from other provinces, especially the northern provinces, or the territories, I should say, they would actually have to go through a two week isolation after our program, so that they can return to their communities. And during that, like we would do our best to stay connected or send them with things to do or journaling to do and such. But they were isolated in a hotel room with one other person. And those times seemed really, really rough on them. Because then they are teams would be gone from their homes for a month. So it kind of also added to their anxieties about coming, because they knew they would have to isolate after coming.

**Moderator 1** 23:35

Right? Yes, certainly a, I would say a major stressor for a lot of families. And like you said, it's it could be a barrier. It's it's already a big commitment. But it's another thing, another hoop to jump through, I guess we have one comment here. I'll just read it out. I really, really wish my child had connected with the weekly calls. But he didn't it was very hard to get him on the call. I would have liked him to continue his connection. But he wasn't interested. This was of no fault to the ranch staff, but just his own issues around phone connection. I also want him to stay connected with this cohort. But that was difficult without violating privacy. Yeah, I think that really gets at some important themes there. I'm wondering maybe XXX or XXX Just a follow up from a clinical staff perspective. When there's instances actually, maybe I should just take a step back and say what when those phone calls? I think it's the I think it's an evening times. Do you kind of encourage children our lessons to have that ongoing communication or is it just really left open up to the child? Yeah, I'd be curious to know just just briefly.

24:52

**Moderator 2**

Yeah, so um, this this participant it has been a while since since they're they're trying have been at the program just because we actually are currently working on quite a change with the weekly calls. But previously, what we had done is that every week, in the evening time, for about half an hour, there was a line that was just like open for any kids who wanted to call into the line. And I was the one to chat with the kids. And it was super fun to just connect and, and it was always up to the families whenever they wanted to call some kids called every week, some kids didn't, or never calls. But we definitely would always encourage that connection.

**Moderator 1** 25:41

Thank you for providing that feedback. I think I will move on to the next question. One second here.

25:53

**Moderator 2**

I can just answer the question there. So just how it's changed. So because of COVID, we had to rearrange a bunch of the different cohorts, because we were only able to take in certain locations at a time. So we were really only able to take in local families. So that really caused a huge change in a lot of our cohorts. So we had to stop the line for a little bit. And we're hoping to do more of like a, a video call with with different kids and to have it more formally set up. And we're kind of working on the structure for that. Because before it was quite more open and more generalized. But now we're hoping to go into more of a of a formalized structured, like zoom session, if that makes sense. Right. Yeah, we think it's a good idea to I think it'll be more engaging for the kids.

**Moderator 1** 26:59

Yeah, thanks for providing that clarification. I think, you know, that's really sounds like it's kind of a scale up from the existing resources, just understanding how critical the family environment is, and to people to have that ongoing communication, even during treatments. That's, that's great to hear. Okay, actually, we have another comment here. So just do that before go to the next question. Our daughter has come a long way since joining Little Warriors, and there's been a lot more peace. Big thanks to you. All working with her. We notice through that she's still struggling with empathy. But we hope this being her final round will help her to come around Fingers crossed. COVID times yes. Our girl has been super lonely most times here at home. But she is now in school making a couple friends. So she is really looking forward to her socialization with her schoolmates. Yeah, that's, that's really good to hear. Thanks for providing that feedback. And yeah, I think I will move to this question here. Now. It's it's a bit of a shift. But the question is, what can we do to improve specifically? And what I really mean by this is, you know, if you had to if you had the opportunity to provide feedback for the clinical team, even government funders, or maybe there's a family that is considering enrolling their child in this Little Warriors treatment program, is there anything that you'd say? Or is there any specific areas that you would you would want for for scaling up and more resources? I'm wondering, yeah, XXX and XXX, did you want to just briefly speak to this? I know last time, we touched upon improvements in collaboration with school counselors, offering tours, a Little Warriors, those sound like really important things. Maybe we'll actually one thing that we didn't touch upon is challenges related to barriers and cultural challenges. I know that some of the programming includes Indigenous elders, for example, or having those kinds of practices available. I actually don't know too much about it. So I'm wondering, could you maybe briefly just speak to that and just explain how that relates to treatment of analysis?

**Moderator 3** 29:36

Yes, so we are connected to an Indigenous elder through Enoch. And while we've had to pause our sweats, due to that just because it's not unfortunately COVID friendly at this time, we were connected with him and it's really, I think, provides such a huge connection for people within In their being able to reconnect with their culture in a healing way. Because we're not just doing Western techniques, we're also we're seeing, and we're recognizing that traditional techniques are also important and validating that for our clients, as well as our staff. And then we also have a grandmother that comes on site during the weekends, and she comes out for one day, and she'll do an Indigenous lead craft for our kiddos. And again, it's teaching them about their culture, or about the culture that we are living on, right, we're living on their land. And so it just, I think, brings awareness and brings respect back to, yeah, brings it back to the land and to the people that we're living with. And, again, like a lot of our kiddos, if they're not connected to their culture, this is a way to kind of dip their toes in as well. And a lot of them are generally very excited to, to share their culture, if they are part of that culture, or to get to know the culture.

**Moderator 1** 31:06

That's great to, to have that space where, you know, culturally informed cares is provided. And that's really it. Yeah, that's really interesting to hear from the kids perspective that they're open. I'm excited to learn. Looks like we have a couple comments here. Government funding for the ranch. Yeah, that's, that's a good piece of feedback there. Thank you. Yeah, I'm not sure XXX or XXX, if you want to speak to that, but I'll just go through some of these comments briefly to Yes, I was super excited to hear about Indigenous elder coming to the camp. But I was sad, it could not happen with with COVID. Yeah, so hopefully, things can open up again and have that inclusion because it does really sound valuable. Even just as a learning piece. Let's see here. I cannot imagine improving the program was amazing. And I'm so very grateful for the opportunity to participate. My son was crippled with his PTSD and taking the shame out of his assault, letting him know that it was possible to move past it, it was invaluable. The changes I've seen over the last few years have impressed me, as I think got better after we left the family family connection that came after the video calls, etc. Thank you to all of you. I'm glad the ranch was able to operate without government funding as it as it was made quite a bit more flexible and responses. Yeah, thank you for providing that feedback. I think just being able to hear some of those lived experience pieces, it's it can be really powerful. And from my student perspective and research perspective, to be able to, again, just increase my own understanding, and how I can better understand the treatment context. Thank you. I think I will move to the next question. Just looking at the time here. I know we're we're actually past the 30 minute mark. So I just wanted to make that notes. I know, we only scheduled for 30 minutes, I'm actually happy to stay on up to an hour in case you have more feedback coming. But if there's anyone in the audience, any of my co host co moderators, if you need to leave by all means. I don't want to keep you so. Yeah, I'll just I'll just keep going, though, to continue. This would be the second last question. So this one, it's, it's more so to do with the beginning of the program. The question is, how was the intake process for you into the program? That is was it easy? Was it difficult? Some of the things that we touched upon last session was the fact that it can be emotionally draining at times, you know, can be exhausting going through a lot of the paperwork. Other individuals, they're very happy to have a tour. Sounds like some were even able to see the cabins and to say goodbye to their child. But maybe for this question. Be also curious to know, the intake process. Maybe XXX or XXX, you've seen differences from that first initial intake, and then that intake for round two. I don't know if a lot of the kids they tend to be more than nervous, sad for both or if you kind of see a trend where you know kids get a bit more comfortable. Maybe you can just briefly speak to that.

34:59

Yeah, There's definitely a lot of nerves a mixed bag of emotion on the first intake day definitely. And that's why it's kind of more of a longer day for the families just because we do take our time to make sure that they have that warm handoff from their kids. I definitely noticed when the kids program then them quite a bit more excited to come back for second, third and fourth round. I think in the team, I don't want to speak for you XXX, but I think in the teen program, it might be a little bit different. There might be some anxiety coming back, just with peer peer relations stuff can be a little bit more intense in the teen program, I think.

**Moderator 3**

Yeah, so going off what XXX was saying, generally, first, first round is quite nerve racking high anxiety. They don't know us they, so they don't have trust in us, which is completely valid. And then, for round two, they're generally round two and three, they're generally excited to come back. But our teens tend to exchange social media information while they're on site. So they do generally continue to talk while they're off site. And through that, sometimes there's some conflicts are dramas that come up being teen girls, and yeah, so that does sometimes play a role in how they're feeling. But we always talk to our families before intake and always ask us the question we always ask is, how is your team feel about coming back and like, they're pretty straight up. And if they need a conversation before coming back to let them know, like, hey, like, when you're here, you're here, you have to leave everything kind of where it's at. And we can resolve things. We can talk through things, then they're usually more willing to come back around for it's quite a bittersweet time. They're generally always very grateful and show a lot of gratitude. And then they're also really excited for the graduation portion. But then they're they know that they're going to miss their cohort members in the staff.

**Moderator 1** 37:23

Right, yeah. Thanks for giving that brief overview. That's, that's really interesting to know. It looks like we have one question. I'm just going back here a bit. How do I get the modules? If I haven't seen any I think, correct me if I'm wrong. I think this is getting to Be Brave bridge. But I'm, I'm actually not sure XXX or XXX, is that something that you have to register through the Little Warrior sites? Or is it just available, open to the general public?

37:51

Yeah, it's open to the general public. I know that when we were slowly launching the program, the referrals went through either myself or XXX and we did it sort of indirectly. But now, you can actually submit a self referral through our website, which is really, really cool. And if this participant is already enrolled in the program, and is having difficulty accessing the modules, I can connect with them and direct them on how to get assets access to those for sure.

**Moderator 1** 38:27

Perfect. Thanks for leaving that open. XXX. It looks like we have a couple other comments here. So I'll just read them out. So excited to be accepted. It was overwhelming with the stacks of paper. The drop off was hard no tour for us because it COVID But it sounds like they felt comfortable and supported. Round one specifically, our daughter wanted to go home. That was really hard, but she ended up working through it and stayed. That's that's really great to hear. Another actually a couple comments here, I was relieved to have found something anything that might help him he was having such a hard time before I found the ranch. The paperwork was nothing compared to the difficulty before that. I was very happy to be able to show him around on the tour beforehand. However, the day I had to leave him was very hard. He had been having such a hard time for so long, but I had a tough time leaving him anywhere. Nevermind with strangers. He also called me every day for the first three weeks begging me to pick him up. And that was really hard emotionally. However, knowing there wasn't much help elsewhere. I made him tough it out. And I'm glad I did. Because this program saved him from whatever might have happened otherwise. Thank you for sharing that. Feedback. I'm really glad to hear that the treatment was something that was providing hope in your life. I'm sure that's a theme that comes up in your conversations. XXX and XXX just to Yeah, that feeling of relief that there is a kind of support out there that is specific to individuals who have who have had child sexual abuse this unfortunate experience. Sounds like this one comment is specifically on the graduation side. The graduation was bittersweet. On one hand, he was ready to move on. But it was hard for me to let go of the support I had to come to rely on. Yeah, I think that that really touches on important theme that we talked about last time that ongoing support after the even the final discharge, I think, yeah, things like peer support, those would definitely be opportunities to, to hopefully scale up for the future. Because we did yeah, we do understand that that's definitely an important determinant of maintaining sustaining treatment outcomes. So your thank you for providing that feedback. I think I will. If there's nothing else here, feel free to continue posting. But I'm going to move to the last question. And the last question is, what did you or your child find most helpful at the time a Little Warriors. So for the first session, some of the things we talked about was the fact that multimodal therapy is used. There's so many different kinds of options available at Little Warriors that they find that children, adolescents, they can kind of gravitate towards ones that they like, or the fields, it's a good fit, or they feel it's really helpful for them. One of those specific comments came up, and it was the fact that Little Warriors has specialized therapists specifically knowledgeable in child sexual abuse, they understand the challenges with this specific context. So that can be very helpful. Yes, so as you start to think of things that your child found most helpful, I'll just open it up to Kailyn. XXX, maybe? Yeah, any kind of changes during COVID? I'm curious, have there been more opportunities to provide virtual options? Or maybe just in general changes during COVID? Were any helpful? Or were they maybe more frustrating is kind of hard to adjust and adapt? I'd be curious to know if you could speak briefly to that.

42:35

**Moderator 2**

Yeah, I think that's something that our team had always wanted to create was something like our online programs for the parents and caregivers, as well as for the kids in the teens as well. So that was always on our radar. And then there, there really wasn't a better time, then COVID To really get going on that and really focusing on that. And it sort of allowed our team to really focus in and build this program out that we think is amazing. And is being used by so many families of ours to which is so great. And now, not only can our families connected to the ranch, access it but some kids and parents out in the community access it, and they feel so supported by the online program that they actually don't feel that their kid needs to go to the ranch. So that's, I mean, that was a very big plus, that came out of COVID was our ability to turn a lot of our supports virtual in that way. And then it's just kind of come such a long way. And we're really proud of it.

**Moderator 1** 43:48

Absolutely. Yeah, thanks for mentioning that. It sounds like one of the comments there. What they found helpful was the staff, they were kind, responsive, observant, understanding and still firm and what they expected from my child. programs are only as good as the staff that run it from another participant top notch therapy, caring and knowledgeable staff, again, on the clinical staff side. So that's that's great to hear. Thanks for providing that. I know one of the things that I chatted about with with the clinical director a Little Warriors was the fact that trauma informed therapy, it's it's an implemented maybe there's some in the audience that don't really or they're not too familiar with this concept. I'm wondering, could you briefly just touch upon that either XXX or XXX, just maybe what that is. And actually, I think there is even a training session for clinical stuff, if I'm not mistaken, but maybe just discuss that role and how it relates to engaging with with children and adolescents?

44:57

**Moderator 2**

Yeah, so um, there's there's a few things for sure. So I'm every staff that comes to work at the ranch, we're all trained in what's called therapeutic crisis intervention training. It's called Tci. For short. So it's offered through Cornell University, and it's a four full day training. So it's quite an intensive training. And if if some of you have heard of the nonviolent crisis intervention training, it's, it really builds even further on top of that, so just really, really allowing us to work with the kids who come through the program, to to just found them to strategize with them to really prevent any, any crises from from happening in the first place, and just supporting them in that way. And then all throughout our time at the ranch, we're always getting trained in different types of trainings. So trauma, trauma one on one, I think just the structuring of the program itself is really trauma informed, of course, and just having that long period of time, especially for that first round of treatment for the kids, just really allowing them I think XXX pointed out earlier was just so that we can meet the kids where they're at, and they can warm up to us as as they feel comfortable to do. So we know some kids take a really, really long time to warm up and and we get that and that's what trauma does to our nervous systems. Right? So we really want to give them that long period of time to just ease into it, because you can't rush that healing.

**Moderator 1** 46:37

Right. Yeah. Thanks for Thanks for mentioning that. It looks like another comment here. Just came in. It says I was glad to hear that when there are times of her daughter calling home crying about when there was a disagreement with another cohort that they're able to work through. And the following day, they were they would call and say that everything was fine. She was okay to stay. Yeah, so glad to hear that. I think that comment and the previous two regarding the staff. And I think that really speaks to XXX, what you just said, there's, there's a lot of training that goes on constantly being educated and being getting more experience. And that's, that's really, that's really the scale of Little Warriors, and the kind of the quality of care that they that they deliver. So yeah, that's, that's really great to hear.

47:32

**Moderator 2**

And XXX, sorry, I meant to mention too, and I don't know if our participants know, but actually, every staff at the ranch, they're assigned their own therapist at the ranch to check in with and, and they're expected to have those individual check ins with we call it their pod lead. So we just really, really value staffs mental health and their being able to ground themselves to then ground to your kids nervous systems. Right. So that's, that's another piece. I don't think we mentioned in the last session.

**Moderator 1** 48:04

Yeah, that that's actually very interesting to hear. Thanks for mentioning that. I think it sounds like it kind of relates to our discussions on, you know, just self care, and boundaries. You know, when you yourself as a clinical staff, for example, you're able to cope well and manage your emotions. Well, you will ultimately be more attuned to be, you know, emotionally present for the children, adolescents. So that's, that's really good to hear. So yeah, I'm still happy to field any more questions, comments, concerns? But that's, yeah. Okay. One question here. So yeah, I'll just, I'll just read that actually. Thankful for this program. I've looked for programs that could help my child, and there wasn't any nearby, but I came across this and got a call back right away. The interview was kind and empathetic, it wasn't rushed. And I felt listened to and I'm very appreciative of the staff and XXX. That's yeah, that's really great to hear. Thanks for providing that feedback. Yeah, so as, as I was just saying, um, yeah, happy to continue the ongoing conversation. I'm just going to go to the last slide. So yeah, if you have any other questions or comments that come up, even after the session, I am always happy to have that ongoing conversation with you. That's my email just right there. So feel free to jump down. But of course Little Warriors is also available to to have that ongoing conversation with you. So yeah, we're just we're just really thankful to be able to partner with you. And thanks again for attending. We're really grateful for all the feedback you provided. So yeah, I think we're, we're just before the hour mark. So I think I'm going to leave it there. Unless there's more. But yeah, thank you again, thank you to all my co hosts and CO moderators for helping with this discussion. And yeah, happy to continue chat. But I think for now, we'll leave it there and hope you have a great evening. So thank you.

**Moderator 2** 50:26

Thank you. Take care. Thank you, XXX.

**Moderator 3** 50:32

And thank you to all the participants that were able to attend tonight. You guys are so helpful to get information from

**Moderator 1** 50:41

Absolutely, yeah. Have a good night, everyone.
